# miRNA-23b-5p affects the proliferation, migration and invasion of osteosarcoma by targeting TMEM127

**DOI:** 10.1007/s12672-022-00519-9

**Published:** 2022-08-08

**Authors:** Dan Zu, Qi Dong, Jun Yao, Sunfang Chen, Bin Fang, Jun Ma, Bing Wu

**Affiliations:** 1 Central Laboratory, The Central Hospital Affiliated to Shaoxing University, Shaoxing, 312030 China; 2 Department of Spine Surgery, The Central Hospital Affiliated to Shaoxing University, Shaoxing, 312030 China; 3 Department of Neurology, The Central Hospital Affiliated to Shaoxing University, Shaoxing, 312030 China

**Keywords:** miR-23b-5p, Osteosarcoma, Migration, Invasion, Proliferation

## Abstract

**Background:**

Osteosarcoma (OS) has become one of the highest mortality cancers in the world due to its late diagnosis, rapid metastasis and rapid recurrence. MicroRNAs can regulate a variety of signaling pathwas involved in cancer development, such as cell proliferation, apoptosis and migration.

**Objective:**

In this study, we studied the biological effects and molecular regulation of mir-23b-5p on human osteosarcoma cells.

**Methods:**

The proliferation of mir-23b-5p in osteosarcoma was measured by CCK8 method and EDU method. In addition, the target population was screened through the database, and the luciferase reporter gene was used to determine the association between miRNA and target gene TMEM127. We verified this result by Western blot.

**Results:**

We found that mir-23b-5p promotes the progression of osteosarcoma by regulating TMEM127.

**Conclusions:**

The results of this study show that mir-23b-5p affects the proliferation, metastasis and invasion of OS by targeting TMEM127.

**Supplementary Information:**

The online version contains supplementary material available at 10.1007/s12672-022-00519-9.

## Highlights

- Survival rates remain very low in patients with osteosarcoma (OS).
- Identification of new diagnostic biomarkers and therapeutic targets is essential.
- miR-23b-5p was transfected into OS cells to study the biological effects.
- miR-23b-5p promoted cell proliferation and metastasis in OS by targeting TMEM127.
- miR-23b-5p may provide diagnostic value in the treatment of OS.

**Supplementary Information:**

The online version contains supplementary material available at 10.1007/s12672-022-00519-9.

## Introduction

MiRNAs are a group of endogenous non-coding RNAs, which mainly regulate gene expression after mRNA transcription [1, 2]. MiRNAs play multiple roles in regulating cell proliferation, apoptosis, cell cycle, metastasis, angiogenesis, and metabolism [3, 4]. There is increasing evidence that microRNAs (miRNAs) play an important role in osteosarcoma (OS) [5]. For example, it has been reported that overexpression of miRNA-206, by targeting Notch3, accelerates the proliferation and metastasis of osteosarcoma cells, thereby accelerating their malignant progression [6]. In addition, Liu et al. found that miRNA-98-5p inhibits osteosarcoma progression by regulating cell cycle progression through downregulation of CDC25A [7]. Current studies have found that the miR-23b-3p/CCNG1 pathway can affect the biological activity of OS cells [8]. However, the biological function of miR-23b-5p in OS has not been well investigated until now.

Transmembrane protein 127 (TMEM127) is a little-known tumor suppressor gene. TMEM127 has been reported to be closely associated with pheochromocytoma, paraganglioma and renal cancer [9]. For example, Stutz et al. found mutations in the TMEM127 gene in pituitary tumors and paragangliomas [10, 11]. Gupta s et al. showed that TMEM127 changes are associated with the development of pheochromocytoma (PC), paraganglioma (PGL), and renal tumors. As many as half of patients with PC/PGL and renal tumors have TMEM127 abnormalities [12]. In addition, sun et al. showed that miR-17-92 cluster could affect gastric carcinogenesis by targeting PTEN or tmem127 to activate Akt/mTOR pathway in gastric cancer. However, the study of OS in TMEM127 has never been reported.

In this study, we compared the expression levels of miR-23b-5p in normal osteoblasts and osteosarcoma cell lines. We analyzed the functional experiments of miR-23b-5p in OS cells. Furthermore, we explored whether TMEM127 was a downstream functional regulator involved in miR-23b-5p exerting its effects on OS cells.

## Materials and methods

### Cell culture

The human Osteosarcoma cell line 143b, Hos, Sao2, U-2OS, HEK-293T were kindly provided by Department of Orthopaedic Surgery, Sir Run Run Shaw Hospital. All cells were maintained in DMEM (Hyclone, USA) supplemented with 10% FBS (ExCell Bio, Jiangsu, China). Cells were incubated in a humidified atmosphere at 37 °C and 5% CO_2_.

### Transfection

MiR-23b-5p inhibitor or miR-23b-5p mimic were transfected into 143b and Hos cells, respectively. Cells were transfected according to the instructions of Lipofectamine 3000 (Invitrogen, Carlsbad, California). Cells were starved for 1 h before transfection. Twenty-four hours after transfection, cells were harvested for further experiments.

### Quantitative PCR analysis

The total RNA of osteosarcoma was extracted according to the instructions of the RNA rapid extraction kit (YISHAN, Shanghai, China). Second, configure the reaction system according to the reverse transcription kit (TIANGEN, Beijing, China). Finally, using a fluorescence quantitative master mix kit (TIANGEN, Beijing, China). The relative gene expression was calculated by using the 2–ΔΔCt method. The bulge-loop RT primer and specific for miR-23b-5p were designed and synthesized by RiboBio(RiboBio, Guangzhou, China)ΔΔCt = ΔCt1–ΔCt2; ΔCt1 represents the difference between the Ct value of the target gene in the test sample and the Ct value of the internal reference gene in the same sample, and ΔCt2 represents the Ct value of the target gene in the control group and the internal reference gene Ct value difference in value.

### CCK-8 assay

Plate the transfected 143b and Hos cells (2 × 10^3^ cells) into a 96-well culture plate with 100 µL of cell suspension per well. Add 10 µL of CCK-8 reagent (GLPBIO, American) to each well at 0, 24, 48, and 72 h, then incubate for 3 h. Measure the absorbance at a wavelength of 450 nm with a microplate reader (ThermoFisher, MA, USA).

### Wound-healing assay

Plate the transfected 143b and Hos cells (3 × 10^5^ cells) into 6-well culture plates. Change to serum-free medium after cells adhere and scrape with a 200 µL pipette tip. Photos were taken at 0 and 24 h, respectively. Finally, the analysis was performed with Image J software.

### Cell invasion assays

Seed transfected 143b and Hos cells (3 × 10^4^ cells^)^ into the upper chamber containing 70 µL of 1 mg/mL matrigel (BD Biosciences, MA, USA) and add 0.6 mL complete medium containing 10% FBS to the lower chamber. After 48 h of incubation, remove cells on top of the membrane with a cotton swab. Cells that migrated to the bottom wells were fixed in methanol for 20 min and stained with 0.1% crystal violet solution. The number of invading cells was counted in three randomly selected light microscopy fields.

### EDU

Cells were transfected with mir-23b-5p mimic/inhibitor overnight. Add 2X EdU (Beyotime, Shanghai, China) working solution and continue to incubate the cells for 2 h, remove the culture medium and add 1 mL fixative solution to fix for 15 min. Then add 1 mL of permeabilization solution and incubate at room temperature for 10–15 min, and wash the cells 3 times. Add 0.5 mL Click reaction solution to each well, incubate in the dark at room temperature for 30 min and wash 3 times. Finally, Hoechst 33,342 was used for nuclear staining. Fluorescence detection can then be performed. Hoechst 33,342 is blue fluorescence with a maximum excitation wavelength of 346 nm and a maximum emission wavelength of 460 nm.

### Dual-luciferase reporter assay

The dual-luciferase reporter plasmid was purchased from Han Bio (Han Bio, Shanghai, China). HEK-293T cells were cultured in 6-well plates at a density of 1 × 10^5^ cells/well. Cells were co-transfected with a plasmid mixture with or without the mir-23b-5p 3′-UTR (500 ng) and TMEM127 3′-UTR or negative control (NC) (10 nM final concentration) using Lipofectamine RNAiMAX (Invitrogen). Luciferase activity was measured after 48 h with a dual-luciferase reporter gene detection kit (Beyotime, Shanghai, China).

### Western blot analysis

Protein samples were collected using lysis buffer containing a protease/phosphatase inhibitor cocktail (Beyotime, Shanghai, China). Proteins were separated using SDS-PAGE and transferred to PVDF membranes (BioRad, AL, USA). After blocking with western blot blocking solution for 1 h, TMEM127 (Immunoway, USA) and β-actin(Dawen, HangZhou, China) primary antibody were incubated overnight. The next day the membrane was incubated with the secondary antibody for 2 h. Proteins were detected using a chemiluminescence kit (Sangson Biotech, Shanghai, China) according to the manufacturer’s instructions. Each experiment was performed in triplicate.

### All statistical analyses were performed using

All statistical analyses were performed using *Graphpad* Prism 6 software. A two-sided P-value < 0.05 was considered statistically significant. Experiments were replicated at least three times and data were shown as mean ± SD in bars.

## Results

### MiR-23b‑5p is up-regulated in osteosarcoma cell lines

**Fig. 1:**
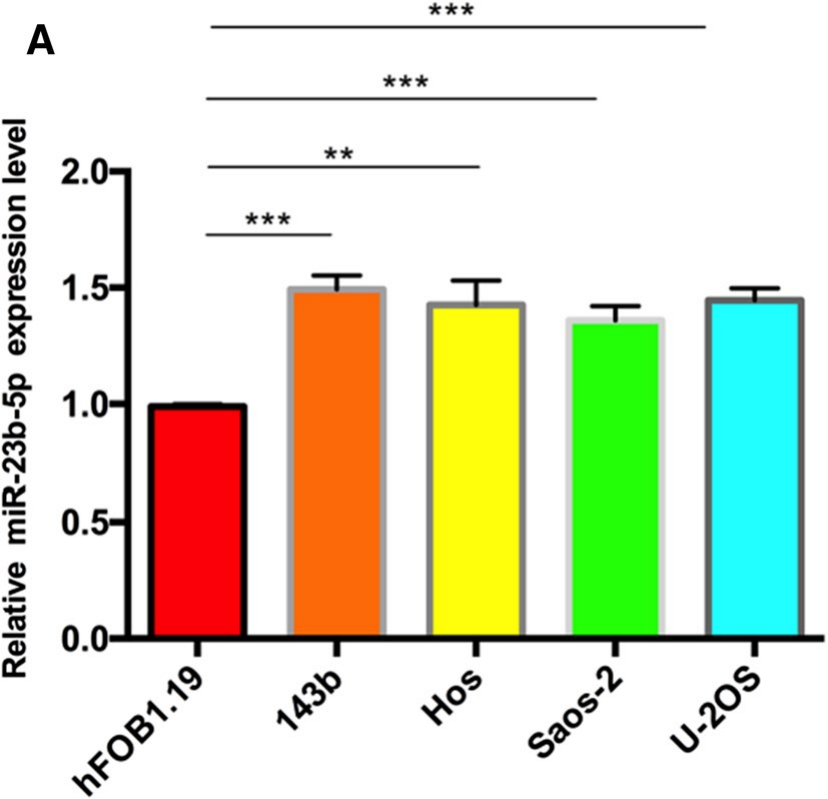
The expression of miR-23b-5p was increased in osteosarcoma. **A** qRT-PCR detection of miR-23b-5p expression levels in osteosarcoma cell lines. Results are shown as mean ± SD from three independent experiments. **P < 0.01

We observed that the expression of miR-23b-5p in the four OS cell lines (U-2OS, 143b, Hos and Saos-2) increased significantly compared with normal osteoblast hFOB1.19. Among them, 143b has the highest expression level, while Saos-2 has a relatively low expression level (Fig. 1A).

### MiR‑23b‑5p promoted cell proliferation, migration and invasion in OS cells

**Fig. 2:**
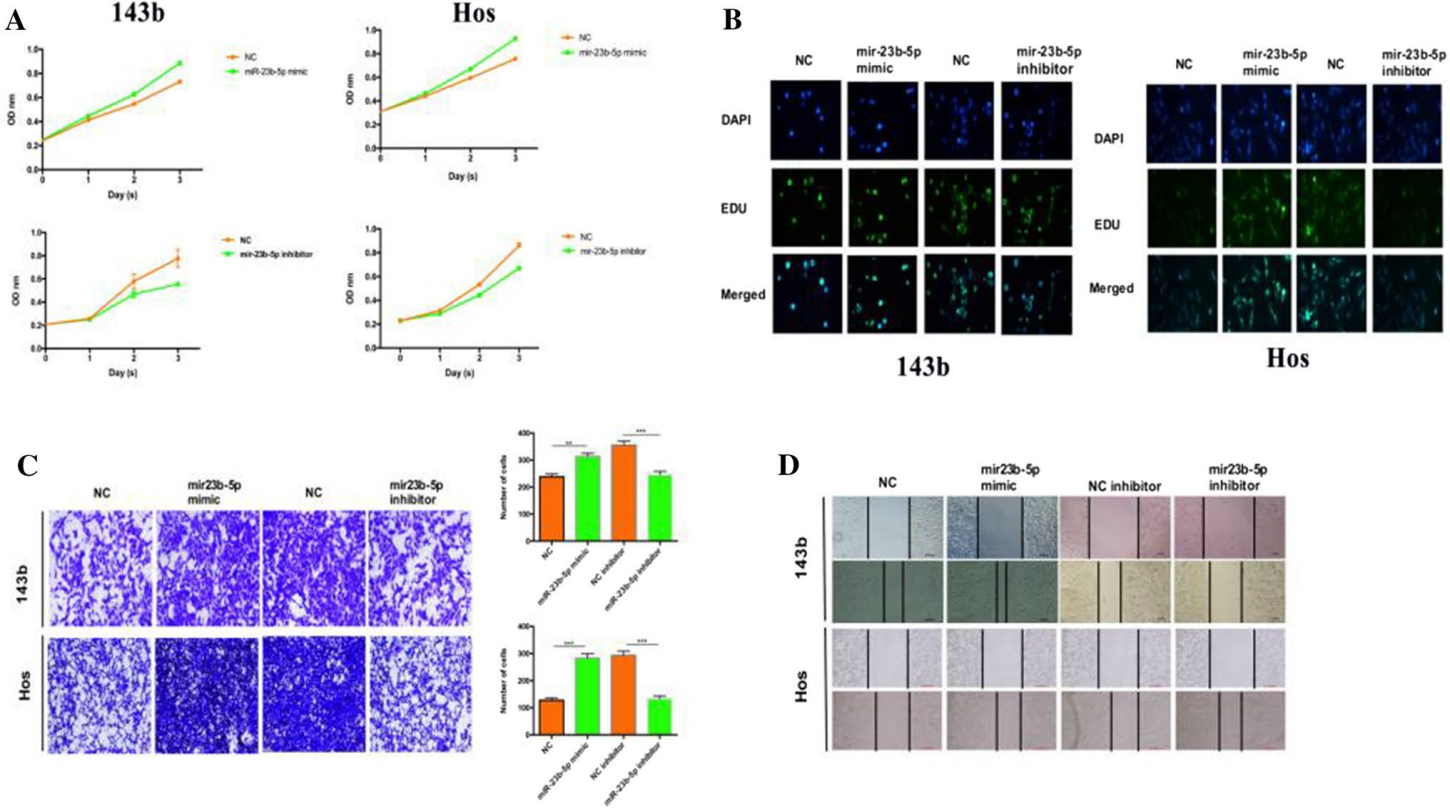
miR-23b-5p promotes proliferation, migration and invasion of osteosarcoma cells. Cell biological functions were assessed by transfection of OS cell lines with miR-23b-5p mimic or inhibitors. **A** Cell viability of 143b and Hos after miR-23b-5p mimic/inhibitor was detected by CCK-8 assay. **B** Cell proliferation kit to detect OS cell proliferation changes. **C** Transwell assay to detect the invasive ability of OS cells. **D** Analysis of OS cell migration ability by wound healing assay. Results are shown as mean ± SD from three independent experiments. **P < 0.01

Here, we transfected miR-23b-5p mimic/inhibitor into 143b and Hos cells. We detecting transfection efficiency using qRT-PCR (Figure S1A-B). CCK-8 and EDU results showed that miR-23b-5p mimic significantly promoted cell viability and proliferation, while miR-23b-5p inhibitor inhibited cell viability and proliferation (Fig. 2A, B). Transwell and Wound-healing assay found that the overexpression of miR-23b-5p promoted the metastasis and invasion ability of osteosarcoma cells, while miR-23b-5p inhibitor inhibited the metastasis and invasion ability (Fig. 2C, D).

### TMEM127 acts as a direct target of miR‑23b‑5p

**Fig. 3:**
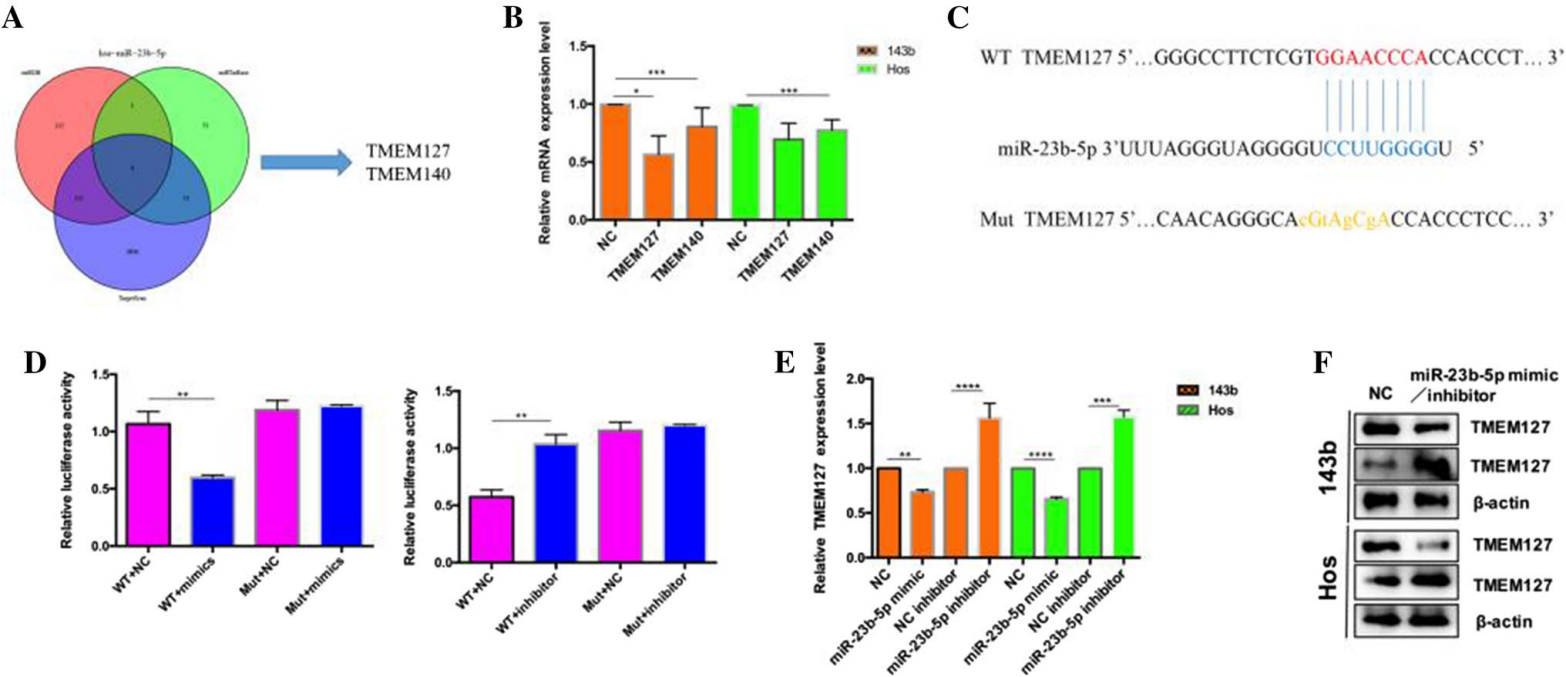
TMEM127 is a direct target of miR-23b-5p. **A** Screening of target genes by intersection of three databases (miRDB, miRBase, and TargetScan). **B** qRT-PCR assessment of the expression levels of potential target genes in OS cells. **C** Schematic diagram of the base sequence between miR-23b-5p and TMEM127. **D** hHEK-293T cells were transfected with miR-23b-5p mimic or NC and wild-type or mutated TMEM127 3′-UTR and subjected to luciferase assay. **E** The mRNA level of TMEM127 in OS cells was assessed by qRT-PCR. **F** The protein expression level of TMEM127 in OS cells was assessed by western blot. Results are shown as mean ± SD from three independent experiments. **P < 0.01

We used the databases TargetScan, miRDB and miRBase to predict the hypothetical target of miR-23b-5p to explore the potential targets of miR-23b-5p in OS cells (Fig. 3A). Based on the qRT-PCR results, TMEM127 was selected for further analysis (Fig. 3B). The results of the luciferase reporter gene test confirmed the direct relationship between miR-23b-5p and TMEM127 3’UTR (Fig. 3C). The results showed that miR-23b-5p knockdown elevated the luciferase activity of the plasmid expressing WT TMEM127, while miR-23b-5p overexpression attenuated the luciferase activity of the plasmid expressing WT TMEM127(Fig. 3D). Furthermore, qRT-PCR and western blot analysis revealed TMEM127 is a true miR-23b-5p target (Fig. 3E, F).

### TMEM127 acts as a tumor suppressor gene in OS

**Fig. 4:**
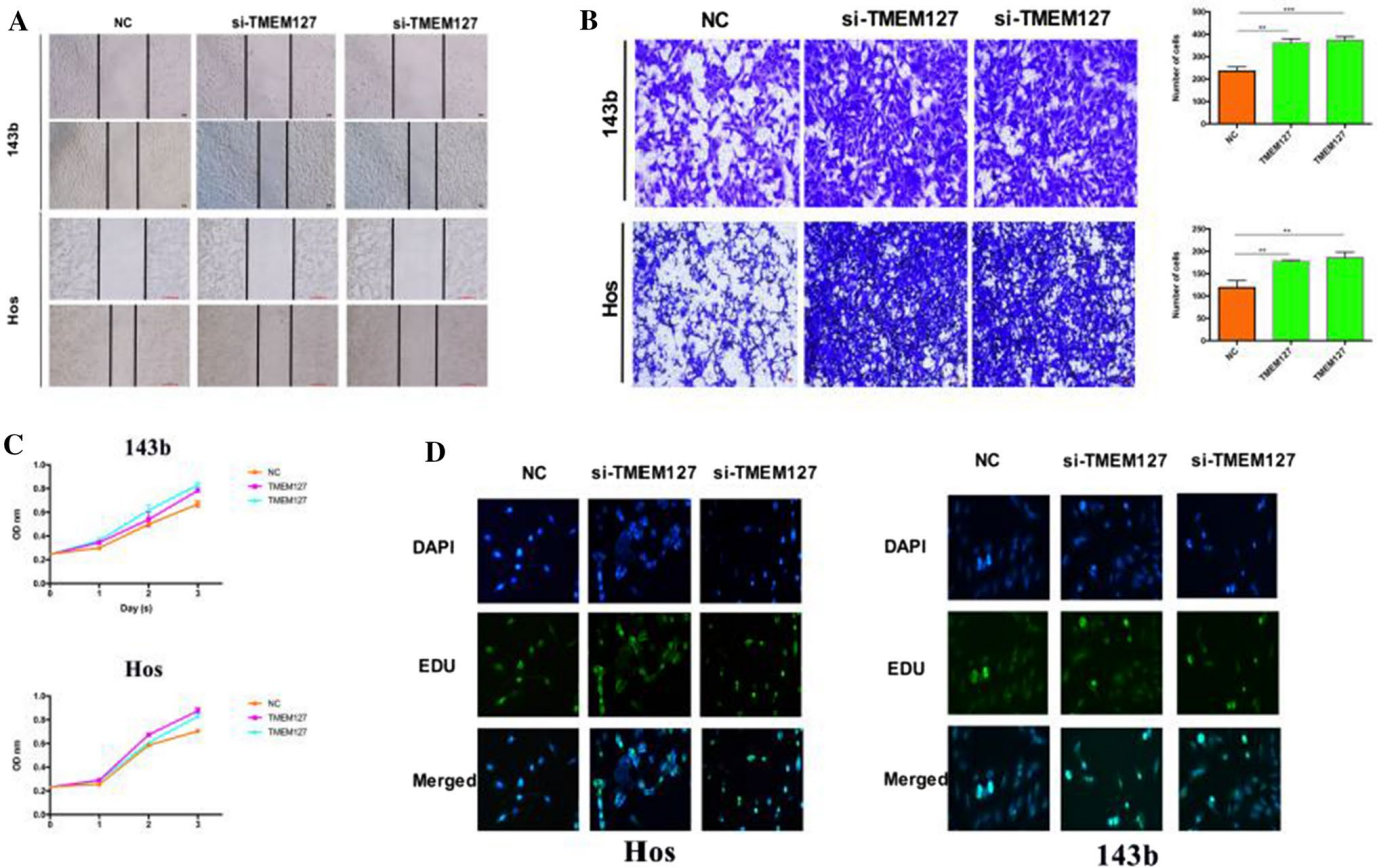
miR-23b-5p promotes OS progression through TMEM127. **A** The migration ability of OS cells was analyzed by wound healing assay. **B** Transwell assay to detect the invasion level of OS cells. **C** Cell viability of 143b and Hos after transfection of siTMEM127 detected by CCK-8. **D** Cell proliferation kit to detect the proliferation ability of 143b and Hos cells after transfection with siTMEM127

It has been reported that TMEM127 acts as an oncogene in various cancers, but its role in OS is unclear. In order to explore the relationship between TMEM127 and OS, we transfected 143b and Hos cells with si-TMEM127 to evaluate their functions in vitro. We detecting transfection efficiency using qRT-PCR (Figure S1C). Wound healing tests and transwell experiments showed that lower levels of TMEM127 promoted osteosarcoma cell metastasis and invasion (Fig. 4A, B). On the other hand, compared with the control group, when the expression of TMEM127 was down-regulated, OS cell viability and proliferation increased significantly (Fig. 4C, D). In summary, our research results show that TMEM127 is involved in the progress of OS.

### TMEM127 is a direct target of miR-23b-5p and is considered an oncogene in OS

**Fig. 5:**
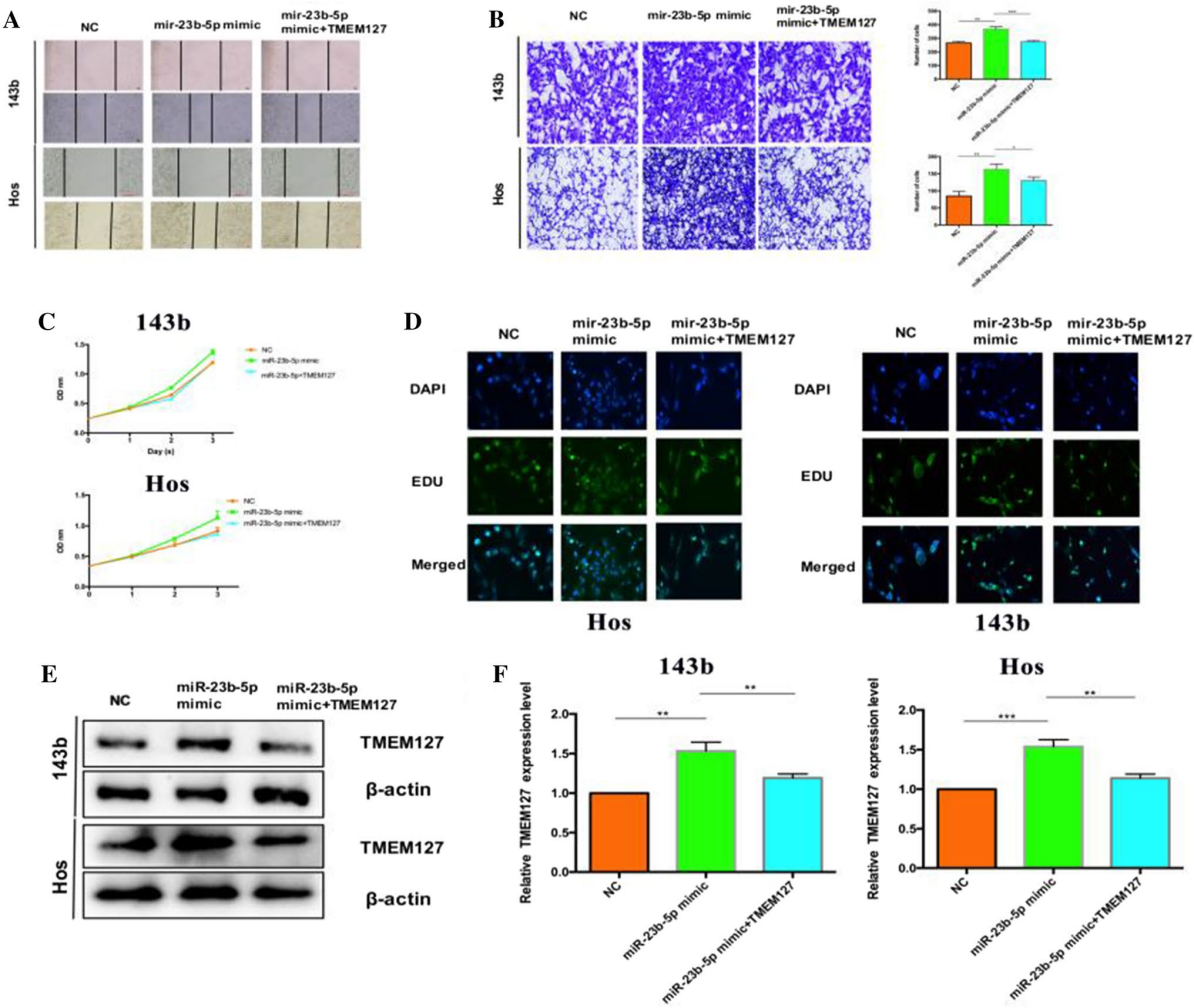
miR-23b-5p targets TMEM127 to promote OS progression. **A** The metastatic ability of cells co-transfected with miR-23b-5p and TMEM127 was detected by wound healing assay. **B** The invasive ability of cells co-transfected with miR-23b-5p and TMEM127 was detected by transwell assay. **C** CCK-8 detection of cell viability after co-transfection of miR-23b-5p and TMEM127. **D** The cell proliferation kit was used to analyze the changes in the proliferation ability of OS cells. **E** The protein expression levels of TMEM127 in OS cells after miR-23b-5p transfection alone and miR-23b-5p co-transfection with TMEM127 were detected by western blot. **F** The mRNA expression levels of TMEM127 in OS cells after miR-23b-5p transfection alone and miR-23b-5p co-transfection with TMEM127 were detected by qRT-PCR. Results are shown as mean ± SD from three independent experiments. **P < 0.01

The TMEM127 overexpression plasmid was constructed and transfected into 143b and Hos cells to further study whether miR-23b-5p affects the progression of OS by targeting TMEM127. Through cell migration and transwell experiments, it was found that high expression of TMEM127 can rescue the down-regulation of miR-23b-5p deficient cells (Fig. 5A, B). In addition, the colony forming ability and cell proliferation ability as assessed by CCK8, overexpression of TMEM127 can improve the down-regulation (Fig. 5C, D). Protein level and gene level also verify this phenomenon (Fig. 5E, F). In summary, these findings indicate that miR-23b-5p is involved in the progression of OS by targeting TMEM127.

## Discussion

In the current study, we first demonstrated that the expression of mir-23b-5p in OS cell lines was significantly upregulated compared with that in normal cells. Functional experiments found that mir-23b-5p mimic promoted the proliferation, metastasis and invasion of OS cells. Mir-23b-5p inhibitor inhibited the proliferation, metastasis and invasion of OS cells. These results collectively suggest that mir-23b-5p may play an oncogene role in the progression of OS, which is inconsistent with its suppressive role in other cancers. For example, mir-23b-5p is downregulated in glioma tissues and cell lines, and overexpression of mir-23b-5p inhibits cell proliferation of glioma cells [13]. Yang et al. found that mir-23b-5p also exhibited a downregulation trend in HCC tissues and cell lines, and lower expression of mir-23b-5p was associated with more severe tumor size and worse survival [14]. These opposing roles of mir-23b-5p in the aforementioned cancers may be attributed to different tissue resources and tumor types. On the other hand, Zhang et al. found that overexpression of mir-23b-3p suppressed HMGB2 expression in PC cells and affected cell proliferation, invasion and apoptosis. Based on these evidences, we confirmed the carcinogenic effect of miR-23b-5p in OS cell behavior.

TMEM127 tumor suppressor gene is a transmembrane protein with unknown coding function [9, 15, 16]. We predicted the targeting relationship between miR-23b-5p and TMEM127 through the database, and verified it through luciferase reporter gene detection. The study found that miR-23b-5p mimic reduced the expression of TMEM127 in OS cells, while miR-23b-5p inhibitor enhanced its expression. We further found that TMEM127 overexpression reversed the increase in proliferation, migration and invasion of 143b and Hos cells induced by miR-23b-5p mimic. Here, we determined that the target gene TMEM127 is a direct downstream regulator, involved in the promotion of miR-23b-5p in OS.

In summary, we first demonstrated that miR-23b-5p is up-regulated in OS cell lines and is a unique marker for OS patients with poor prognosis. Functionally, we further proved that miR-23b-5p is a promoter of OS cell proliferation, migration and invasion by targeting TMEM127, which provides a potential diagnostic marker and therapeutic target for the treatment of osteosarcoma.

## Supplementary Information

Below is the link to the electronic supplementary material.

Supplementary file1 (pdf 236 KB). S1: Transfection efficiency. (A) qRT-PCR detects the transfection efficiency of miR-23b-5p mimic. (B) qRT-PCR detects the transfection efficiency of miR-23b-5p inhibitor. (C) qRT-PCR detects the transfection efficiency of TMEM127

## Data Availability

The datasets used and/or analysed during the present are available from the corresponding author on reasonable request.
