# Supplementary material for: miRNA-23b-5p affects the proliferation, migration and invasion of osteosarcoma by targeting TMEM127
Source: Discov Oncol. 2022 Aug 8;13:71. doi: 10.1007/s12672-022-00519-9 (PMC9360392; doi:10.1007/s12672-022-00519-9)
Supplement: Supplementary file 1 — Supplementary file1 (pdf 236 KB). S1: Transfection efficiency. (A) qRT-PCR detects the transfection efficiency of miR-23b-5p mimic. (B) qRT-PCR detects the transfection efficiency of miR-23b-5p inhibitor. (C) qRT-PCR detects the transfection efficiency of TMEM127 [file 12672_2022_519_MOESM1_ESM.pdf]

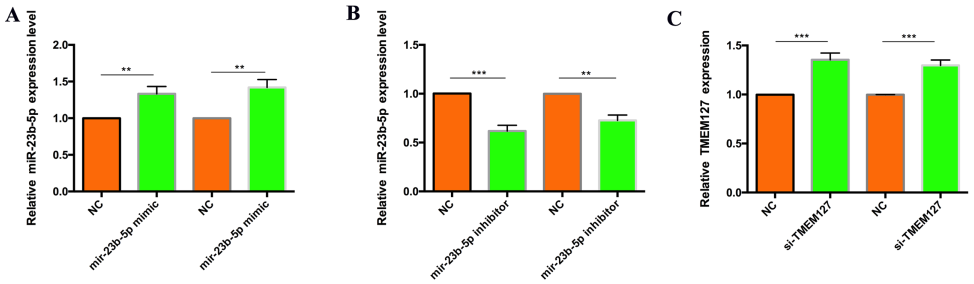

S1: Transfection efficiency. (A) qRT-PCR detects the transfection efficiency of miR-23b-5p mimic. (B) qRT-PCR detects the transfection efficiency of miR-23b-5p inhibitor. (C) qRT-PCR detects the transfection efficiency of TMEM127.
